# *Chromolaena odorata* layered-nitrile rubber polymer transdermal patch enhanced wound healing in vivo

**DOI:** 10.1371/journal.pone.0295381

**Published:** 2024-03-11

**Authors:** Mazlyzam Abdul Latif, Asrul Mustafa, Lee Chee Keong, Asmah Hamid

**Affiliations:** 1 Faculty of Health Sciences, Center for Toxicology and Health Risk Studies (CORE), Universiti Kebangsaan Malaysia, Jalan Raja Muda Abdul Aziz, Kuala Lumpur, Malaysia; 2 Faculty of Health Sciences, Biomedical Science Program, Universiti Kebangsaan Malaysia, Jalan Raja Muda Abdul Aziz, Kuala Lumpur, Malaysia; 3 Latex Science and Technology Unit, Malaysian Rubber Board (MRB), MRB Experimental Station Sungai Buloh, Selangor, Malaysia; COMSATS University Islamabad - Lahore Campus, PAKISTAN

## Abstract

The objective is to investigate the healing efficacy of a *Chromolaena odorata* layered-nitrile rubber transdermal patch on excision wound healing in rats. Wounds were induced in Sprague-Dawley rats and were later treated as follows: wound A, the negative control, received no treatment (NC); wound B, the negative control with an empty nitrile rubber patch (NC-ERP); wound C, treated with a *C*. *odorata* layered-nitrile rubber patch (CO-NRP); and wound D, the positive control with Solcoseryl gel with a nitrile rubber patch (PC-SG-NRP). After 1, 3, 6, 10, and 14 days, the rats were sacrificed and analyzed for wound contraction, protein content, hexosamine, and uronic acid levels. Macroscopic observation showed enhanced wound healing in wounds treated with CO-NRP with a wound contraction percentage significantly higher (p<0.05) on days 6 and 10 compared to those treated with NC-ERP. Similarly, protein, hexosamine, and uronic acid contents were also significantly higher (p<0.05) in CO-NRP-treated wounds when compared with wounds treated with NC-ERP. Histological findings showed denser collagen deposition and faster granulation tissue formation in wounds treated with CO-NRP. From the results obtained, it is concluded that the *C*. *odorata* layered-nitrile rubber transdermal patch was effective in healing skin wounds.

## Introduction

Wounds cause discomfort and are more prone to infection than other troublesome complications [1]. Impaired wound healing also leads to significant patient morbidity and mortality [2]. Generally, wound healing is a complex biological process to regenerate a new dermal layer upon injury [3,4]. The wound healing process comprises four complementary and overlapping phases: the hemostasis phase, inflammatory phase, proliferative phase, and remodeling phase [5]. Although there have been some advances in the wound healing process, the best treatment remains to be determined, and the duration cannot be shortened. Many current wound healing treatments are aimed at dry wound healing, such as the topical application of creams, gels or traditional standard bandages used to cover and protect the wound. Nevertheless, the excess wound exudates would be required to be absorbed and the wound be kept from drying. Hence, moist wound healing is an advanced treatment that benefits dry healing. A transdermal patch is one of the healing agents for moist wound healing [6]. Transdermal delivery of wound healing drugs offers an alternative to improving patient compliance and effectiveness. Biomedical advantages of transdermal delivery include the avoidance of systemic first-pass metabolism and controlled release of drugs over an extended period, besides providing a convenient, non-invasive means for systemic and topical drug delivery [7].

Integrating natural products *Chromolaena odorata* and nitrile rubber polymer in producing transdermal patches would become a novel way of wound therapy. *Chromolaena odorata* (family Asteraceae) is a type of weed that grows wild throughout the country on various soils, mainly found in paddy fields and crop plantations. It is a medicinal plant widely used in folk medicine in tropical countries like Thailand and Southeast Asia [8]. The plant is popularly known because the extract of *C*. *odorata* leaves is often applied to fresh wounds to stop bleeding and heal external wounds [9]. Through in vitro studies, the *C*. *odorata* leaves have been described to exhibit potential pharmaceutical properties such as anti-bacterial, antioxidant, promoting hemostasis, and stimulating cell migration and proliferation, which are vital in wound healing [8,9].

Commonly, polyurethane film is used in making the backing layer of the transdermal patch [6]. However, other synthetic rubbers, especially nitrile rubber polymers with many particular characteristics, could also be used as a backing layer for transdermal patches. Nitrile rubber is currently used to produce transparent nicotine transdermal patches, condoms, and examination gloves [10–12]. Nitrile rubber polymer is produced from the copolymerization of acrylonitrile and butadiene [13]. It can be easily manufactured at a meagre cost. Being a synthetic polymer, nitrile rubber does not cause skin allergies and is resistant to many chemicals, such as acids, alkalis, oils, and water. The nitrile rubber patch can physically protect the wound area [14]. Due to its water-resistant property, a nitrile rubber transdermal patch incorporated with *C*. *odorata* can prevent excessive water loss in burn wounds while also maintaining wound exudates and a moist environment in the wound bed, allowing optimum wound healing. Its resilience property also gives it high flexibility for the transdermal patch [15]. Both nitrile rubber polymers and *C*. *odorata* have been proven to have low cytotoxicity and no genotoxic effect [16,17]. Therefore, the unique characteristics of both *C*. *odorata* leaves, and nitrile rubber polymer could be combined to produce a transdermal patch. This patch would be suitable for treating burns and other mild external wounds caused by abrasion, stabbing and tearing. The present study aims to investigate the effect of a *C*. *odorata*-layered nitrile rubber transdermal patch on wound healing in rats. The transdermal patch was prepared and applied to wounded rats, after which wound healing was assessed with a wound contraction percentage. Similarly, the protein content, hexosamine and uronic acid levels as a function of wound healing time were also characterized using spectrophotometry.

## Materials and methods

### Preparation of *C*. *odorata* layered nitrile rubber polymer transdermal patch

The plant was identified by Mr. Mohd Shahidan Mohamad Arshad, programme head and researcher from the natural products division, Forest Research Institute of Malaysia (FRIM). Fresh *C*. *odorata* leaves were washed with distilled water and dried overnight in an oven at 40°C. The leaves were then ground into powder using a pulverizer at a speed of 10,000 rpm, after which they were sieved to obtain particles with sizes less than 1 mm. The resulting powder was then dispersed into nitrile latex, with a concentration of 16 wt%, before pouring into a glass mould. The mixture was then transferred into an oven and dried at 70°C for 30 minutes. Upon drying, the resulting film was compressed using a load of 50 N to form a thin film, washed with methanol, and redried in the oven at 40°C for 2 hours.

### Preparation of rats for testing

All procedures that were performed on rats were approved by the Universiti Kebangsaan Malaysia Animal Ethics Committee (UKMAEC) with approval number: FSK / BIOMED / 2010 / MAZLYZAM / 22—SEPTEMBER/332 –SEPTEMBER—2010 FEBRUARY—2011. A total of 30 female Sprague-Dawley rats weighing 200–250 g each were used for the study. The rats were individually housed in clean polyethylene cages under standard experimental conditions of temperature 23 ± 2°C, 12 h light/dark cycle and fed on a standard pellet diet of water and libitum. The rats were used for the experiment after an acclimatization period of one week.

### Full-thickness excision wound modelling

**Fig 1:**
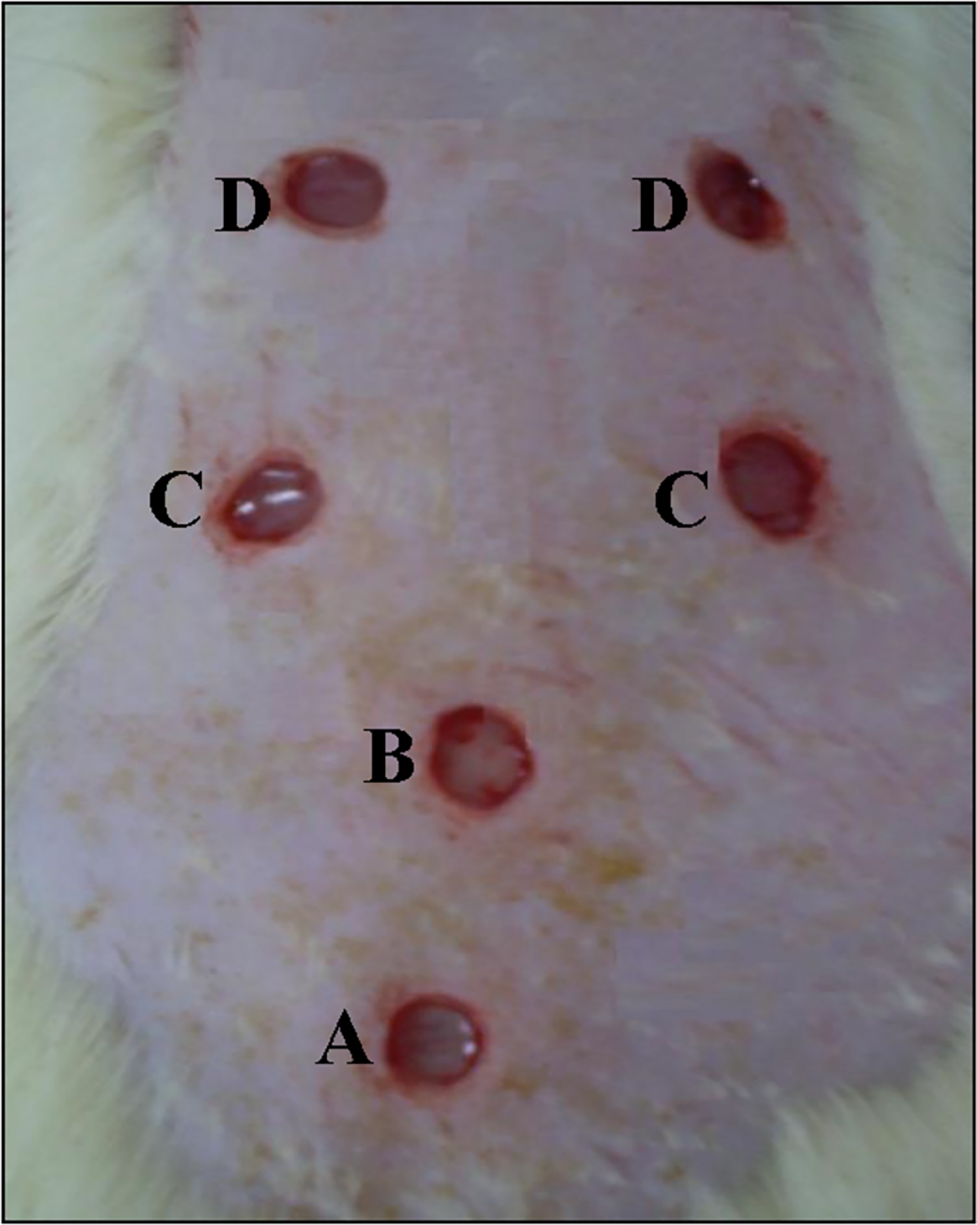
Six excisional wounds were induced on the dorsum of rats on day 0. (I) Wound A, the control, received no treatment (NC). Wound B was the control treated with an empty nitrile rubber patch (NC-ERP). Wound C was treated with a *C*. *odorata* layered-nitrile rubber patch (CO-NRP). Wound D, which acted as the positive control, was treated with Solcoseryl gel and a nitrile rubber patch (PC-SG-NRP).

The rats were put on overnight fasting before being anasthetized with a cocktail of ketamine, xylazil and zoletil intravenously (10 mg/kg body weight). Their dorsal surface was shaved with a sterile razor blade (Topaz, India) and cleaned with 70% alcohol to maintain aseptic conditions. The rats were next randomly divided into 5 groups according to the days they were being sacrificed, namely days 1, 3, 6, 10, and 14, with each sacrifice containing 6 rats. Six full-thickness excisional wounds on a circular area of 36 mm^2^ were created using a 6 mm diameter biopsy punch on the dorsum of each rat, as shown in Fig 1. Wound A (negative control,NC) received no treatment at all. Wound B (negative control) was treated with an empty nitrile rubber patch (NC-ERP). Wound C was treated with *a C*. *odorata*-layered nitrile rubber patch (16% by weight of nitrile rubber) (CO-NRP). Wound D, which acted as a positive control, comprised a combination of Solcoseryl gel with a nitrile rubber patch (16% by weight of nitrile rubber) (PC-SG-NRP). Excess bleeding was cleaned using sterile gauze. The transdermal patches were held in their places with hypoallergenic adhesive tapes and covered with nonwoven cohesive bandages. The treatments and nitrile rubber patches were changed every 2 days. The rats were closely monitored for any infection, and if any positive signs of infection were observed, the rats were then separated, excluded from the study, and replaced. The uninfected rats were sacrificed by sodium pentobarbital (100 mg/kg) injection via intraperitoneal at intervals of the 1st, 3rd, 6th, 10th, and 14th days after wound creation. A wound tissue area of 10 mm^2^ was excised, and its weight was recorded before being stored at -70°C until biochemical analysis. The remaining wound tissues were used in histological processing.

### Macroscopic observation and contraction percentage determination of wounds

Macroscopic observation of wounds was done to determine the wound healing stage. Photographs were taken to compare wound healing between the treated and control groups. The wound contraction percentage was assessed by tracing the excision wound on the day of wounding. Subsequently, the rats were sacrificed on those days using sterile transparent paper and a permanent marker. The wound areas recorded were measured using 1 mm^2^ graph paper. Changes in the wound area, indicating the wound contraction percentage, were calculated using the following equation [18]:

$$Wound\;size\;reduction\;(\%)=[A_{0}–A_{t}]/A_{0}\;x\;100$$ (1)

where *A*_0_ dan *A*_t_ are the wound areas on day 0 and after time interval *t*, respectively.

### Biochemical analysis of excision wounds

#### Protein determination

The protein content of the tissue was determined according to the procedure by Bradford [19]. The wound tissue was first washed with 1.15% potassium chloride, refrigerated at 4°C and mixed with 0.1 M phosphate buffer pH 7.4 at volumes ten times the weight of the tissue. The tissue was then homogenized at 16 000 rpm for approximately 2 minutes before centrifuging at 3,000 rpm at 4°C for 5 minutes. Then, 0.1 ml of the supernatant solution was mixed with 9.9 ml of phosphate buffer, after which a 0.1 ml sample was pipetted out and mixed with 5.0 ml of Bradford reagent. The sample was remixed thoroughly using a vortex mixer and left for 5 minutes at room temperature. The absorbance was then read at 595 nm in a 1-cm cell spectroscopically, and the values were fitted to a calibration curve to obtain the protein content. All results were statistically compared, and the *p-values* were also calculated.

#### Determination of hexosamines

Hexosamines were extracted from the granulation tissues using a modified method of Cheng [20]. The tissues were dried in an oven at 60°C for 75 hours. In test tubes, 20 mg of the dried tissues were mixed with 2.5 ml of 4.0 M HCl. The tubes were then tightly closed and transferred into a water bath with boiling water for 4 hours to hydrolyze the tissues. Upon hydrolysis, the hydrolysates were diluted with distilled water to a total volume of 10 ml before filtering with a Whatman No. 1 filter paper. A total of 2 ml of filtered hydrolysates were neutralized with 2 ml of 1.0M NaOH. The hydrolysates were then determined for their hexosamine. To 1 ml of filtered hydrolysates in test tubes, 1 ml of freshly prepared acetylacetone reagent was added and mixed before boiling in a water bath for 15 minutes. The tubes were cooled under running tap water before being mixed with 5 ml of 95% ethanol. Finally, 1 ml of Ehrlich’s reagent was added, and the resulting mixtures were diluted to a volume of 10 ml with 95% ethanol. The resulting dark pink to purple colour was then read for absorbance at 530 nm using a spectrophotometer. The value was fitted to a calibration curve to obtain the concentration of hexosamines. The results of all groups were statistically compared, and the *p-values* were calculated.

#### Determination of uronic acid

Uronic acid was extracted from the granulation tissues according to the procedure described by Biter and Muir [21]. The tissues were dried in an oven at 60°C for 75 hours, and the resulting dry weight was measured. The tissues were then digested using papain (10 mg/g dry weight of tissue) in 10 of ml 0.5 M acetate buffer, containing 0.005 M cysteine and 0.005 M di-sodium salt of EDTA at 65°C for 24 hours. In separate tubes, 5 ml of sulphuric acid was pipetted and cooled to 4°C before transferring 1 ml of the digested samples. The tubes were then heated for 10 minutes in boiling water and cooled to room temperature. A total of 0.2 ml of carbazole was added, the tubes were shaken again, and heated in boiling water for another 15 minutes before cooling to room temperature. The absorbance of the samples was then read at 530 nm using a spectrophotometer, after which the results were fitted to a calibration curve to obtain the concentration of uronic acid in the samples. The results of all groups were statistically compared, and the *p-values* were calculated.

### Histological observations of wounds

Wound granulation tissues measuring 10 mm (length) x 2 mm (width) were placed on slides and stained with hematoxylin & eosin (H&E) and Masson’s Trichrome. They were then viewed under a light microscope at 100x magnification.

### Statistical analysis

All biochemical experiments were performed in duplicate. Data were expressed as mean ± standard error of mean (SEM). The results were compared statistically by one-way ANOVA using the SPSS (version 27.0) program. The data were considered statistically significant at *p* < 0.05.

## Results and discussions

This study aimed to evaluate the wound healing potential of *C*. *odorata*-layered nitrile rubber patch on excision wounds in experimental rats. The combined use of nitrile rubber polymer and a *C*. *odorata* in a patch was transdermally applied, and the efficacy was compared with the standard commercial formulation of Solcoseryl gel. Transdermal delivery of wound healing drugs offers an alternative to improving patient compliance and effectiveness. The combined use of nitrile rubber polymer and the natural product *C*. *odorata* in transdermal patches will become a novel way of wound therapy, especially for burn wounds. The role of nitrile rubber backing in transdermal patches as a suitable vehicle for delivering *C*. *odorata* must be addressed owing to its wound protection abilities and maintaining an optimum microenvironment suitable for wound healing [22]. The nitrile rubber transdermal patch can be designed in various shapes and sizes, making it suitable to cover a wide range of burned skin areas to prevent significant water loss from wounds.

Previous studies have shown that *C*. *odorata* promotes migratory keratinocyte stimulation, keratinocyte upregulation of extracellular matrix protein and basement membrane component synthesis, and fibroblast protection of collagen lattice contraction. Furthermore, *C*. *odorata* also increased the expression of numerous adhesion complexes, such as laminin-5, laminin-1, collagen IV, and fibronectin, by human keratinocytes. *C*. *odorata* also induced the production of genes such as heme oxygenase-1, thromboxane synthase, and anti-platelet aggregator matrix metallopeptidase 9 (MM9), which increased the hemostatic process and wound healing activity. *C*. *odorata* can encourage the migration and proliferation of fibroblast cells [8,23].

**Fig 2:**
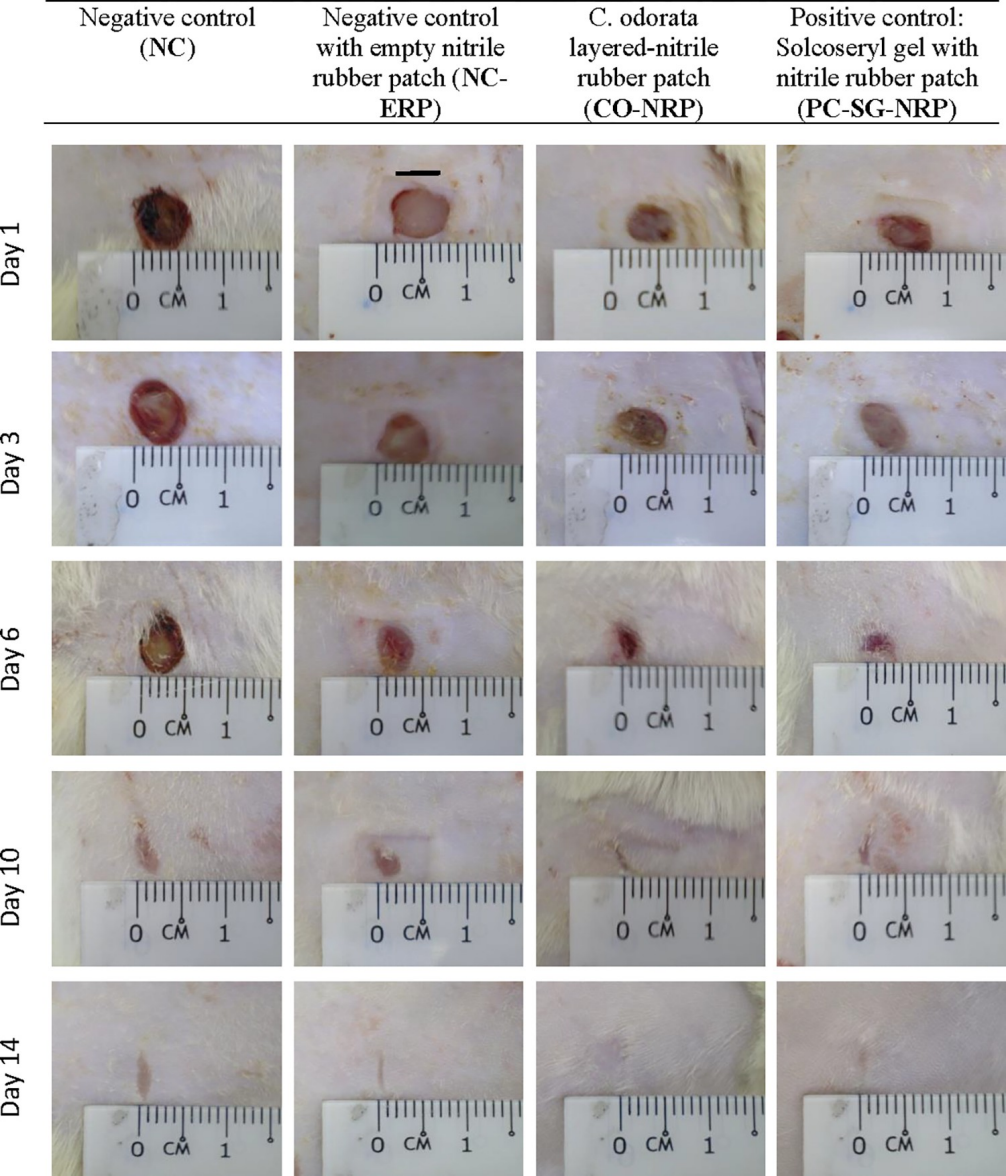
Excisional wound closures in rats as a function of wound treatment and time.

In this study, *the C*. *odorata*-layered nitrile rubber patch’s role in wound healing was investigated by comparing the macroscopic observations of dermal wound healing (Fig 2). All wounds showed significant macroscopic changes on day 6, at which pink-coloured granulation tissue was abundantly formed. In wounds treated with CO-NRP and PC-SG-NRP, 6 mm full-thickness dermal wounds were observed to heal rapidly, with lesions reduced to half within the first 6 days and completely healed within 14 days with colourless smooth scars. Although healing was delayed in a wound treated with NC-ERP since it was only covered with an empty nitrile rubber patch, the lesion size decreased by 2 mm at 6 days and with a higher healing rate than the negative control wound (NC), which was without the nitrile rubber patch. However, both negative control wounds (NC and NC-ERP) were not completely healed at 14 days. A dry, hard, and crusty scab formed on the surface of the negative control wound, whereas the wounds treated with empty nitrile rubber patches only (NC-ERP) were moist. From the results, it was observed that wounds which were covered with nitrile rubber patches healed faster than the negative control wound. This showed that the wounds healed through a moist wound-healing environment with the transdermal patch, which enhanced the wound-healing process. This observation was supported by the research done by Winter, who proved that wounds healed two times faster in moist conditions than when the wound was covered by crusty dry scabs [24]. Observation of the fastest wound area closure in wounds treated with CO-NRP revealed an enhanced wound healing effect comparable to the standard drug Solcoseryl gel.

**Fig 3:**
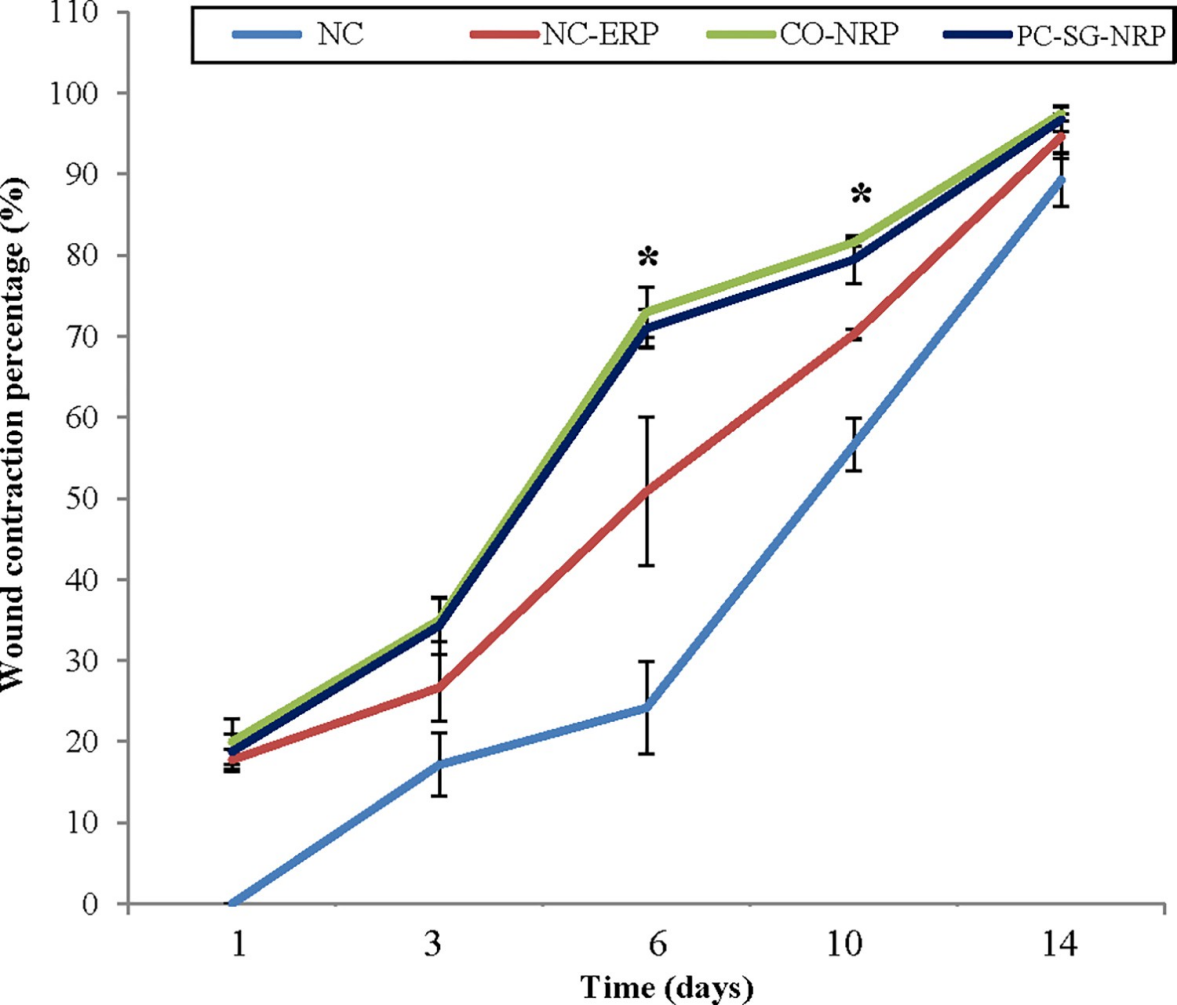
Effect of transdermal patches on the level of wound contraction percentage in an excision wound model. n = 6 rats in each group; values are mean ± SEM, *Significant at p < 0.05 when wound treated with CO-NRP was compared with wound treated with empty nitrile rubber patch (NC-ERP).

Wound contraction is another parameter used to assess wound healing. From the results in Fig 3, wound contraction started immediately on day 1 in all wounds except for the untreated negative control (NC), which only began to contract on day 3. Wound contractions continued to progress until day 14. The *C*. *odorata*-layered nitrile rubber patch significantly enhanced wound closure (CO-NRP) as it showed the highest wound contraction percentage, followed by the Solcoseryl gel nitrile rubber patch (PC-SG-NRP) and empty nitrile rubber patch (NC-ERP). Wound contraction percentage was significantly higher (p < 0.05) in CO-NRP-treated wounds on days 6 and 10 compared to wounds treated with NC-ERP. Although the wound treated with an the empty nitrile rubber patch (NC-ERP) contracted faster than the untreated negative control wound (NC), the effects were statistically insignificant. The wound contraction is the process of the wound area shrinking depending on the tissue’s healing ability, the type and extent of tissue damage and the general state of the health of the tissue [25]. Wound contraction was significantly faster, with the highest wound contraction percentage in the wound treated with a nitrile rubber patch containing *C*. *odorata* (CO-NRP). Compared to the results of Mahmood *et al*., the wound treated with CO-NRP in this study healed faster than the wound treated with a combination of honey and an aqueous extract of *C*. *odorata* leaves, which required 16 days [26]. The improved wound contraction rate of the *C*. *odorata*-layered nitrile rubber patch application may be due to the stimulation of interleukin-8. This inflammatory α-chemokine affects the function and recruitment of various inflammatory cells, fibroblasts, and keratinocytes [18]. It may increase the junctional intracellular communication gap in fibroblasts by inducing a more rapid maturation of granulation tissue. Besides that, there may also be the presence of more myofibroblasts in the wound bed of wounds treated with CO-NRP, which is responsible for wound contraction [25].

**Fig 4:**
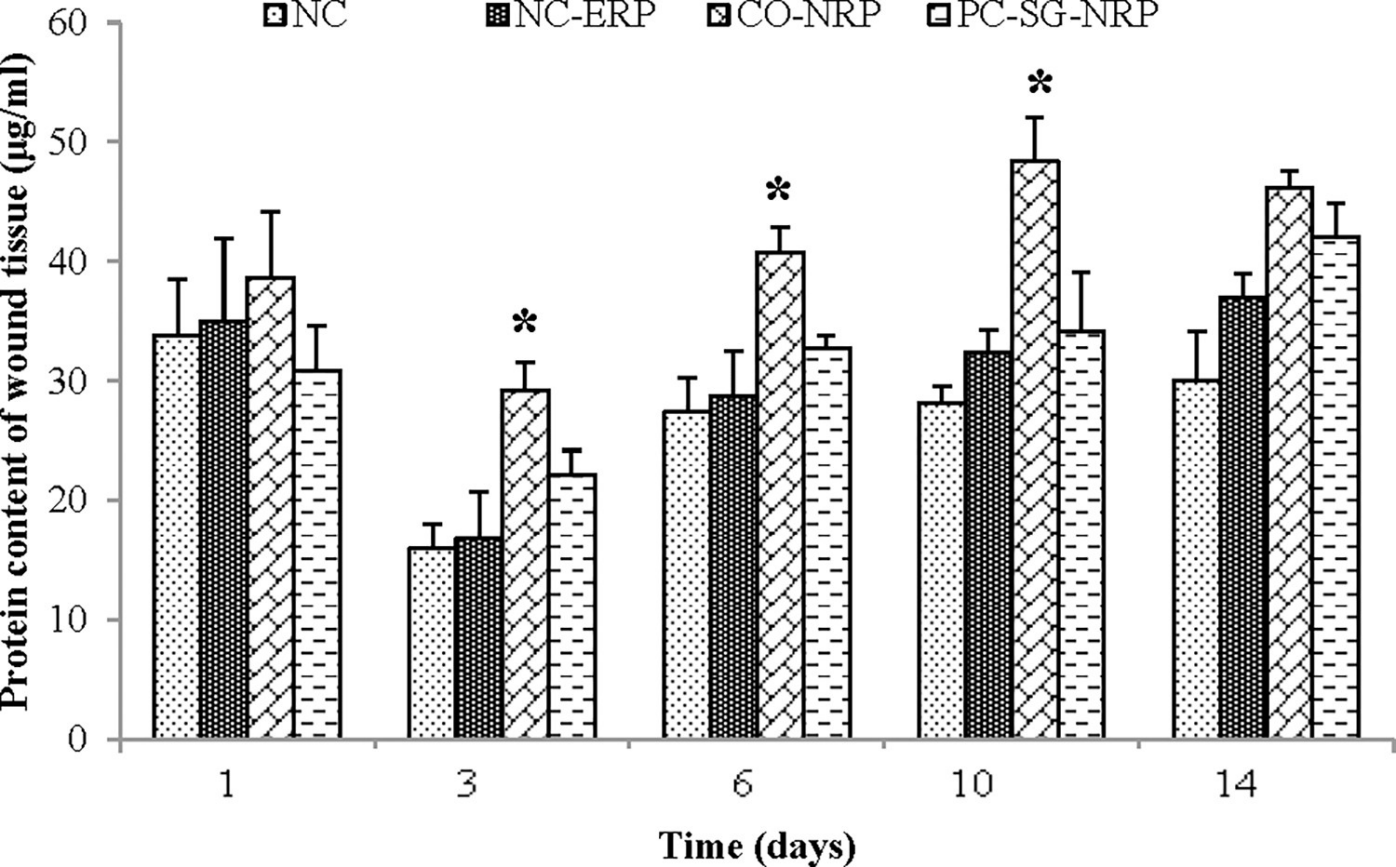
Effect of different treatments on the protein content of excision wounds as a function of time. n = 6 in each group; values are mean ± SEM, *Significant at p < 0.05 when CO-NRP treated wound was compared with NC-ERP treated wound.

The protein contents in the granulation tissues of all untreated and treated wounds are given in Fig 4. High protein content was recorded for all wounds on day 1, which declined on day 3. The protein content, however, increased again, reaching maximum values on day 14. Throughout the study, protein levels were highest in wounds treated with CO-NRP, followed by wounds treated with PC-SG-NRP. Protein levels were lowest in the untreated negative control wound (NC). Protein levels were significantly higher (p < 0.05) in CO-NRP-treated wounds on days 3, 6, and 10 compared to NC-ERP-treated wounds. The protein content of wound tissue represents the overall levels of proteins involved in different wound healing phases and cellular proliferation. Wounds treated with a *C*. *odorata*-layered nitrile rubber patch (CO-NRP) resulted in significantly higher protein content in early wound healing which might be due to the presence of enzymes, cytokines, C-reactive proteins, coagulation proteins, and complement proteins [4]. All these proteins are responsible for promoting inflammation in the wound bed which enhances wound healing compared to the control wound. Furthermore, the *C*. *odorata*-layered nitrile rubber patch (CO-NRP) could also increase cellular proliferation and collagen synthesis at the wound site, as evidenced by significantly higher wound tissue protein content in late healing processes. This is because almost one-third of the total body proteins are collagen, the most abundant protein found in the human body [27]. The increased proliferation rate of fibroblasts, endothelium cells, and keratinocytes would also lead to high protein levels [28]. This is supported by an in vitro study by Phan *et al*. that showed that besides extracellular matrix proteins, *C*. *odorata* extracted from leaves could enhance the proliferation of fibroblasts, endothelial cells, and keratinocytes [29].

**Fig 5:**
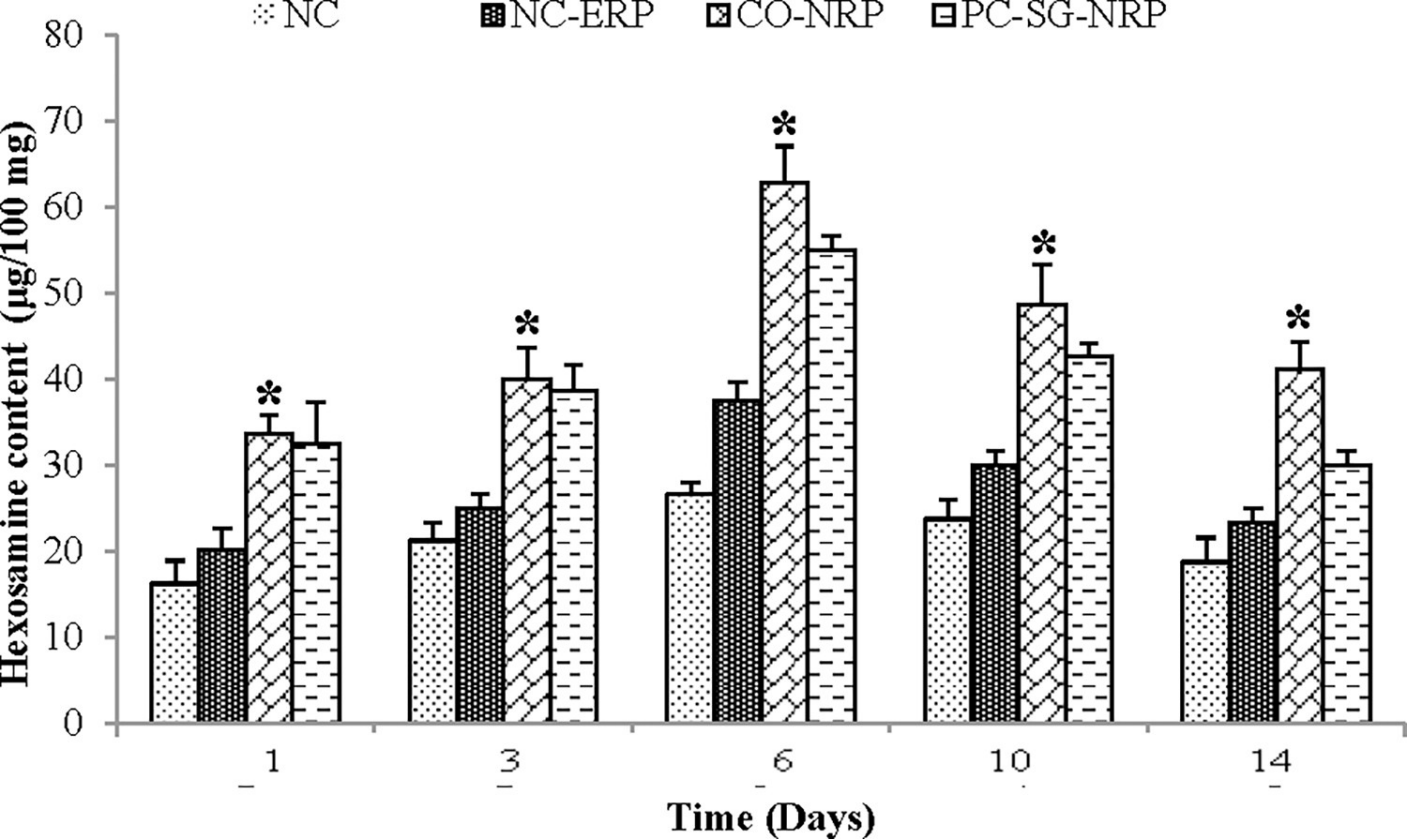
Effect of different treatments on the hexosamines content of excision wounds as a function of time. n = 6 rats in each group; values are mean ± SEM, *Significant at p < 0.05 when wound C was compared to wound B.

The hexosamine content in the granulation tissues of the untreated and treated wounds was analyzed and compared. From the results in Fig 5, it was observed that wounds treated with a *C*. *odorata*-layered nitrile rubber patch showed the highest hexosamine content in the granulation tissues, which was significantly higher (p < 0.05) on all days compared to wounds treated with an empty nitrile rubber patch (Fig 5). Though so, the hexosamine levels in all untreated and treated wounds increased, reaching maximum values on the 6th day, after which it decreased gradually until day 14.

**Fig 6:**
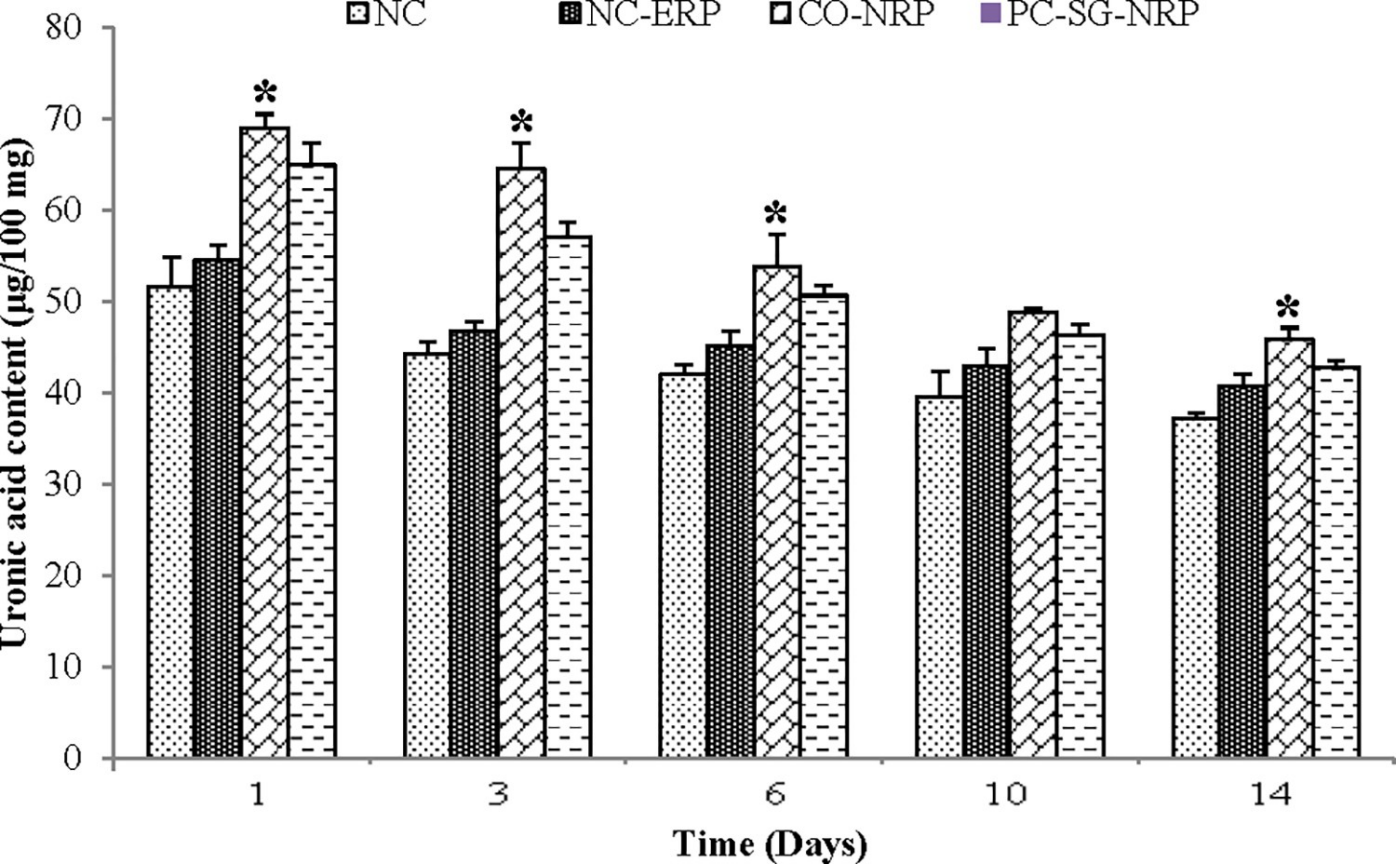
Effect of different treatments on the uronic acid content of excision wounds as a function of time. n = 6 rats in each group; values are mean ± SEM, *Significant at p < 0.05 when wound C was compared with wound B.

Uronic acid levels were determined for all untreated and treated wounds, and the results are shown in Fig 6. Uronic acid levels were highest on day 1 for all wounds, after which there was a constant decrease in their levels up to day 14. Wounds treated with a *C*. *odorata*-layered nitrile rubber patch gave the highest uronic acid content value, which was significantly higher (p < 0.05) on all days when compared to wounds treated with an empty nitrile rubber patch (NC-ERP).

Hexosamine and uronic acid are the vital building blocks of the glycosaminoglycan in the extracellular matrix [30]. Significant increases in hexosamine and uronic acid contents indicate increased glycosaminoglycan synthesis corresponding to increased wound healing. High hexosamine and uronic acid levels were observed in the early part of wound healing, gradually decreasing [31]. Increased hexosamine and uronic acid release into the wound exudates indicate increased glycosaminoglycans synthesis in early wound healing [32]. On the other hand, the decrease in hexosamine and uronic acid was associated with a concomitant increase in collagen content [33]. Glycosaminoglycan like hyaluronic acid protect the granulation tissue from oxygen-free radical damage, stimulating wound healing. The glycosaminoglycan also stabilize the collagen fibers by enhancing electrostatic and ionic interactions by controlling collagen’s ultimate alignment and characteristic size, which help promote wound healing [18].

**Fig 7:**
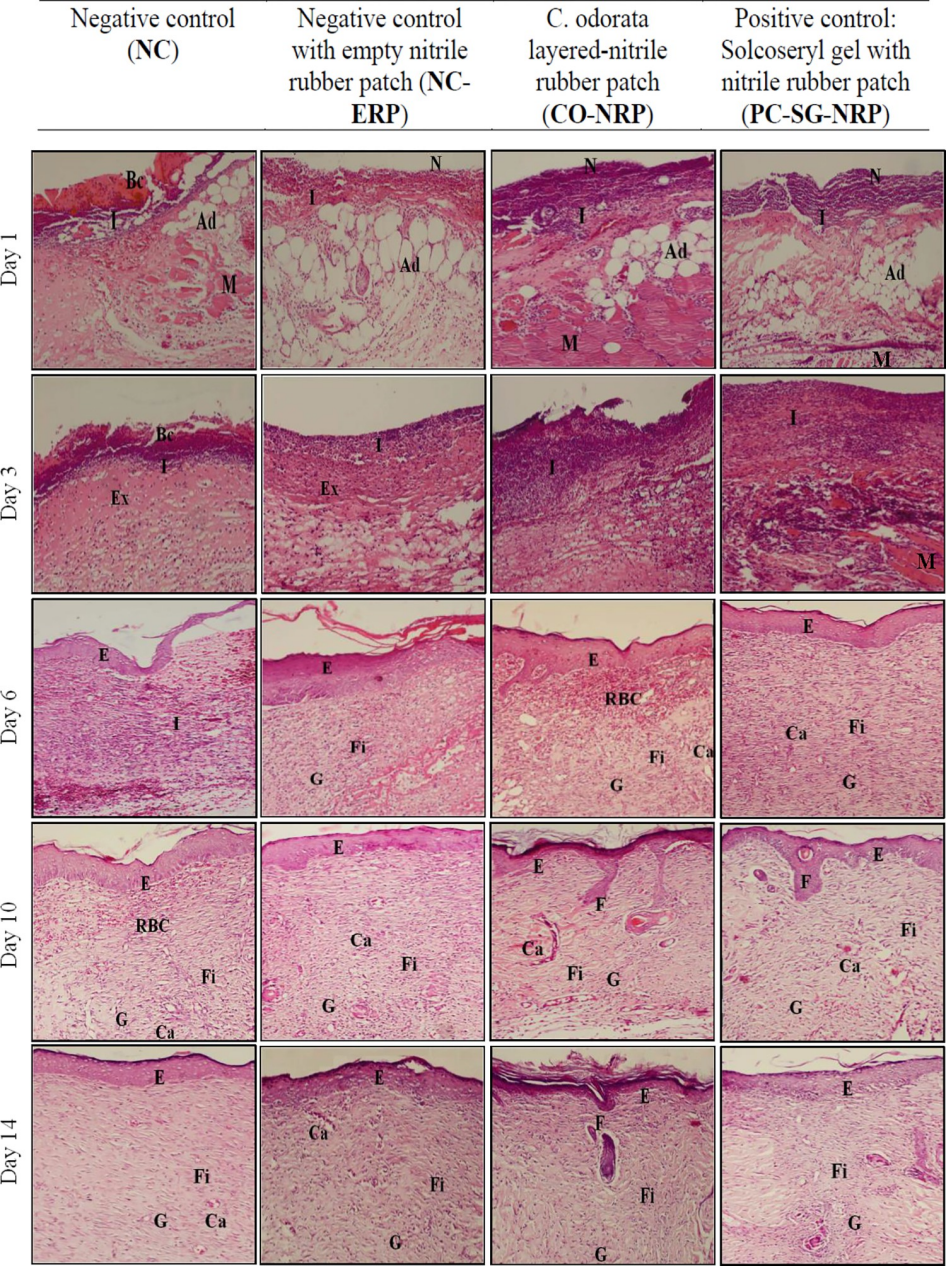
Images of skin tissue sections stained with haematoxylin and eosin show histological changes during the wound healing in untreated and treated wounds as a function of time. Magnification:100x. Note: Ad = adipose tissue, Bc = blood clot, Ca = capillaries, E = epithelium, Ex = exudates, F = hair follicle, Fi = fibroblasts, G = granulation tissue, I = acute inflammatory cells, M = muscles layers, N = necrotic slough, RBC = red blood cells.

Detailed histopathological analysis of H&E sections showed enhanced wound healing in the wound, which was treated with a *C*. *odorata*-layered nitrile rubber patch compared to the untreated wound (Fig 7). On day 1, all wounds except the untreated (NC) exhibited a superficial layer of the wound, lined by a thin layer of necrotic slough. Cell debris, red blood cells with distinct oedema underneath with expanded space, fibrin deposits and scattered polymorphonuclear cells such as neutrophils were observed within the damaged tissue. CO-NRP-treated wounds exhibited larger fibrin-filled spaces and more significant numbers of neutrophils infiltrated than the negative control (NC and NC-ERP). On the other hand, numerous red blood cells and platelet aggregations were observed on the surface of NC wounds, forming a red scab. On day 3, CO-NRP-treated wounds exhibited more fibrinous exudates, while neutrophils and mononuclear cells infiltrated the interstitium more significantly than in NC-ERP wounds. On day 6, the keratinocyte layer regenerated (re-epithelization) at the wound site, covered with the nitrile rubber patch. Fibroblasts were visible in the matrix, and neo-vascularization with numerous new capillaries was evident within the wound site to form granulation tissue. CO-NRP and PC-SG-NRP wounds exhibited more significant migrating fibroblasts and capillaries at the wound site. On day 10, hair follicles regenerated on the CO-NRP and PC-SG-NRP compared to the NC and NC-ERP wounds. On day 14, the granulation tissue in CO-NRP and PC-SG-NRP wounds appeared to mature into a pale collagenous fibrous scar with a distinct orthokeratotic keratinocyte layer. Here, wound healing appeared to be complete. In contrast, NC and NC-ERP wounds were not completely healed by day 14, as indicated by the presence of increasing blood capillaries in the superficial and deeper dermis, which indicated the granulation tissue to be still in the vascular form, and by the presence of mononuclear cells infiltration. The maturation of granulation tissue was in the initial stages.

**Fig 8:**
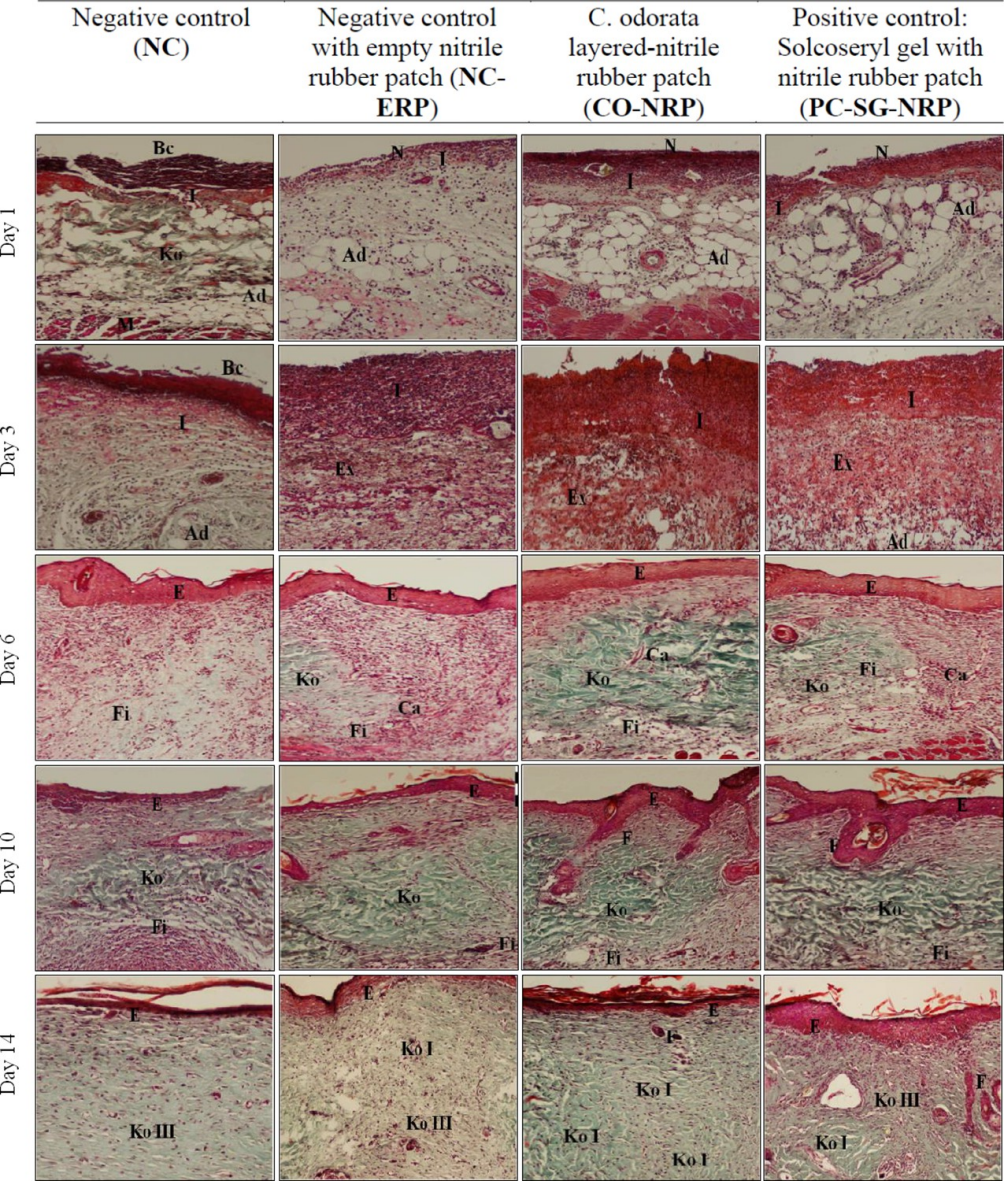
Images of skin tissue sections stained with Masson’s Trichrome showing histopathological changes during the wound healing process in untreated and treated wounds as a function of time. Magnification: 100x. Note: Ad = adipose tissue, Bc = blood clot, Ca = capillaries, E = epithelium, Ex = exudates, F = hair follicle, Fi = fibroblasts, Ko = collagen, Ko I = type-I collagen, Ko III = type-III collagen, I = acute inflammatory cells, M = muscles layers, N = necrotic slough.

Similarly, histopathological analysis of the wound tissues using Masson’s Trichrome stain was also conducted. The results shown in Fig 8 showed that new collagens were not synthesized yet on days 1 and 3. Instead, aged and damaged collagen fibers were being digested by collagenase. However, on day 6, the collagen fibers began to deposit themselves in the matrix of wounds treated with CO-NRP and PC-GS-NRP with a higher packing density in CO-NRP-treated wounds than the other wounds. On day 10, more prominent collagen deposition was exhibited in the dermis (scar tissue) of CO-NRP and PC-GS-NRP treated wounds compared to negative control wounds (NC and NC-ERP), which contained fewer mononuclear cells and numerous distinct blood capillaries. On day 14, collagen fibers were deposited from the wound bed to the superficial layer beneath the epidermis in all wounds. Though so, the collagen fibers present in wounds CO-NRP and PC-GS-NRP were more mature, as indicated by the changes of type-III collagen (arranged linearly) to type-I matured collagen, which was cross-linked with one another to form a more irregular pattern of collagen bundles that strengthen the fibrous scar. The collagen fibers observed in wounds NC and NC-ERP were still type-I collagen.

From the histological observations of wound tissue sections, the H&E staining supported the biochemical measurement results. Higher amounts of acute inflammatory cells and fibrinous exudates were present in the wound treated with a *C*. *odorata*-layered nitrile rubber patch, which suggests the presence of higher biochemical compounds in the wound bed along with other exudates such as extracellular matrix proteins, hexosamine, uronic acid, cytokines, and complementary proteins. Observations of rapid re-epithelialization in wounds treated with *C*. *odorata*-layered nitrile rubber patches support the higher wound contraction percentage than control wounds. High proliferation and large quantities of fibroblasts and new capillaries in wounds treated with a *C*. *odorata*-layered nitrile rubber patch suggest enhanced cellular proliferation, angiogenesis, and denser collagen deposition synthesized by fibroblasts [4,29]. The Masson’ Trichrome staining revealed the presence of high amounts of collagen fibres in the wound treated with a *C*. *odorata*-layered nitrile rubber patch and Solcoseryl gel compared to the control wound. Furthermore, the collagen in the wound bed also acquired faster maturation by transforming to type-I collagen from type-III collagen [34]. This suggests *that C*. *odorata*-layered nitrile rubber patch could enhance and promote wound healing faster than the standard drug Solcoseryl gel.

## Conclusions

It is concluded that the *C*. *odorata*-layered nitrile rubber transdermal patch possesses excisional wound healing properties as depicted by the higher wound contraction percentage and significantly higher increment in the protein, hexosamine, and uronic acid levels in wound tissue, together with histological findings. Hence, the results support the efficacy of the *C*. *odorata*-layered nitrile rubber transdermal patch in treating excisional wounds in rats and could be further developed into a novel wound healing agent for pharmaceutical applications.

### Supporting information files

MTT assay is one of the screening techniques for the cytotoxicity effect of the soluble water solution of nitrile rubber polymer and *Chromoleana odorata* on V79 fibroblast cells. The positive control used for MTT is zinc sulphate (ZnSO_4_). The negative control is an empty NRP patch. The MTT graph is plotted by calculating the percentage of cell viability based on the read absorbent value and compared with the negative control (non-compound media). The percentage value of cell viability is expressed in the average triplicate ± SEM. ANOVA statistical tests are carried out to compare the percentage of viability with the negative control used. For leachate preparation, a total of 0.2g of CO-NRP compound was submerged in 1 ml of DMEM supplemented with 10% FBS (Fetal bovine serum) for 72 hours in the CO_2_ incubator with 5% CO_2_ to produce a leachate solution with a concentration of 0.2g/ml. A series of dilutions were prepared with the leachate solutions with concentrations ranging from 200 μg/ml (highest) and 6.25 μg/ml (lowest). A cell culture plate with V-79 cells (100% confluence) was treated with a series of diluted CO-NRP leachate (200, 100, 50, 25, 12.5 and 0 μg/ml) for 72 hours. MTT assay was performed after 72 hours of incubation.

## Supporting information

S1 Fig Cell viability graph (%) versus CO-NRP leachate preparations at 200, 100, 50, 25, 12.5 and 0 μg/ml. No IC50 value was observed at all leachate concentrations after 72 hours of treatments. The result showed that CO-NRP was safe and did not cause toxicity to the V79 cells. The test was done in triplicate for all the concentrations. All readings were calculated in average ± SEM compared to the negative control (no treatment).
(TIF)
