# Supplementary figures and images for: Chromolaena odorata layered-nitrile rubber polymer transdermal patch enhanced wound healing in vivo
Source: PLoS One. 2024 Mar 11;19(3):e0295381. doi: 10.1371/journal.pone.0295381 (PMC10927106; doi:10.1371/journal.pone.0295381)

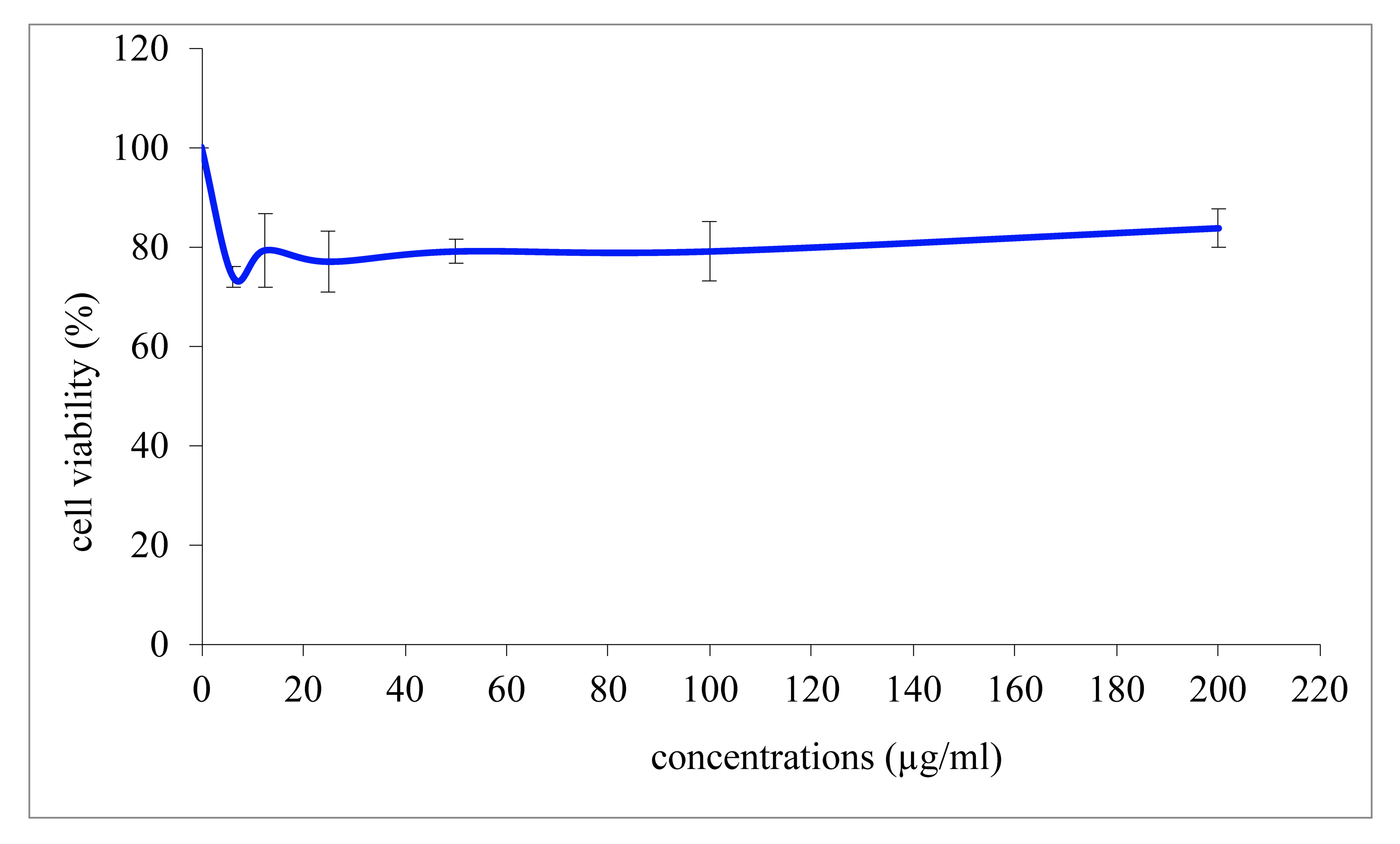

Supplement: S1 Fig — No IC50 value was observed at all leachate concentrations after 72 hours of treatments. The result showed that CO-NRP was safe and did not cause toxicity to the V79 cells. The test was done in triplicate for all the concentrations. All readings were calculated in average ± SEM compared to the negative control (no treatment). (TIF) [file pone.0295381.s001.tif]
